# Enhanced detection of nitrogen dioxide via combined heating and pulsed UV operation of indium oxide nano-octahedra

**DOI:** 10.3762/bjnano.7.144

**Published:** 2016-10-25

**Authors:** Oriol Gonzalez, Sergio Roso, Xavier Vilanova, Eduard Llobet

**Affiliations:** 1 MINOS-EMaS, Universitat Rovira i Virgili, Avda. Països Catalans, 26, 43007, Tarragona, Spain; 2 ICIQ, Institute of Chemical Research of Catalonia, Avda. Països Catalans, 16, 43007, Tarragona, Spain

**Keywords:** dynamic gas sensing, indium oxide, nitrogen dioxide, pulsed UV light, UV-activated metal oxide

## Abstract

We report on the use of combined heating and pulsed UV light activation of indium oxide gas sensors for enhancing their performance in the detection of nitrogen dioxide in air. Indium oxide nano-octahedra were synthesized at high temperature (900 °C) via vapour-phase transport and screen-printed onto alumina transducers that comprised interdigitated electrodes and a heating resistor. Compared to the standard, constant temperature operation of the sensor, mild heating (e.g., 100 °C) together with pulsed UV light irradiation employing a commercially available, 325 nm UV diode (square, 1 min period, 15 mA drive current signal), results in an up to 80-fold enhancement in sensitivity to nitrogen dioxide. Furthermore, this combined operation method allows for making savings in power consumption that range from 35% to over 80%. These results are achieved by exploiting the dynamics of sensor response under pulsed UV light, which convey important information for the quantitative analysis of nitrogen dioxide.

## Introduction

Technological barriers related to sensor performance and power consumption are currently limiting the implementation of widely distributed, smart wireless sensor systems that can be deployed and then remain in operation with no further human intervention. Overcoming these barriers entails developing gas sensors featuring low cost, small size, enhanced sensitivity, selectivity, stability and low power consumption. The task of indoor and outdoor air pollution monitoring would certainly benefit from the implementation of grids of wireless sensing nodes [1]. In particular, radio frequency identification (RFID), has been identified as a widely extended technology in which the integration of one or more gas sensors in a tag, would turn the tag into a wireless sensor that could be easily read with an inexpensive reader via a radiofrequency link [2–3]. Nowadays, distributed wireless boxes to monitor air pollution already exist. These employ electrochemical gas sensors that are ultra-low power and very sensitive. However, such systems do not meet the requirements of personalized or indoor air pollution monitoring because of the high cost and size of the sensors.

In environmental pollution monitoring, the detection of nitrogen dioxide is of particular interest because this pollutant is known to be a source of acid rain and fog, to catalyse the formation of ozone and to trigger diseases of the respiratory system in humans [4–5]. The combustion of transportation fuels is responsible for over 50% of the anthropogenic emissions of nitrogen oxides. Some metal oxide semiconductors have been found to be highly sensitive to nitrogen dioxide levels in air [6–7] and there are commercially available metal oxide NO_2_ sensors [8]. In particular, many authors have reported nanostructured indium oxide as a promising material for the sensitive detection of nitrogen dioxide at trace levels in air [9–12]. However, the facts that metal oxides are known for their lack of selectivity and this, combined with their normally high operating temperatures, have prevented their integration as gas sensitive material in the tags of RFID sensing systems.

However, there have been reports of gas sensors using ultraviolet (UV) activated metal oxides [13–16]. These works employ UV light as an energy efficient alternative to heating for activating chemical reactions occurring at the surface of metal oxides during gas detection. This approach could significantly cut power consumption in metal oxides and, therefore, help re-considering the suitability of these materials for integrating wireless sensors. Integrating gas sensors with UV LEDs would certainly increase the cost of production (LED and packaging). However, these extra costs could be kept at a fraction of those incurred when producing a standard MOX sensor if UV-activated sensors were produced in big numbers. UV light has often been used exclusively for promoting desorption of surface species from the sensing layer, rather than to modify its sensing properties. It is only very recently that UV activation in addition to heating has been explored for improving sensitivity and selectivity of metal oxides [17].

In this paper the use of heating and pulsed UV irradiation is investigated in view of enhancing the sensitivity and lowering power consumption of a resistive, nitrogen dioxide sensor that employs semiconducting indium oxide nano-octahedra as gas-sensitive nanomaterial (a detailed description of the sensor fabrication procedure can be found in the Experimental section). The dynamics of sensor response towards different concentrations of nitrogen dioxide under pulsed UV light activation are presented and discussed. Dynamic response is employed to obtain new response features that correlate well with nitrogen dioxide concentration. The usefulness of the technique is assessed in terms of sensitivity enhancement and reduction in power consumption.

## Results and Discussion

### Morphology and crystalline phase analysis

**Figure 1:**
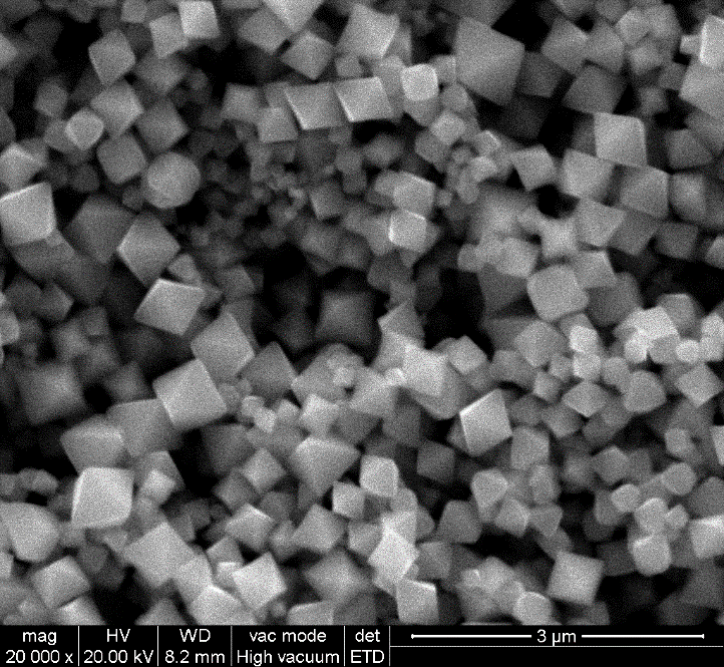
SEM micrograph of as-grown indium oxide nano-octahedra.

SEM was performed to study the morphology of the indium oxide nanomaterial. Figure 1 shows a SEM micrograph of the as-grown material. The image shows that the nanomaterial consists of well-defined octahedral shaped structures. No other morphologies are observed, which indicates the uniformity of the process. Regular octahedra consist of eight equilateral triangles, four of which meet at the same vertex. The size of the octahedra range between 200 and 500 nm. All faces are almost perfectly smooth and without any visible structural defects.

**Figure 2:**
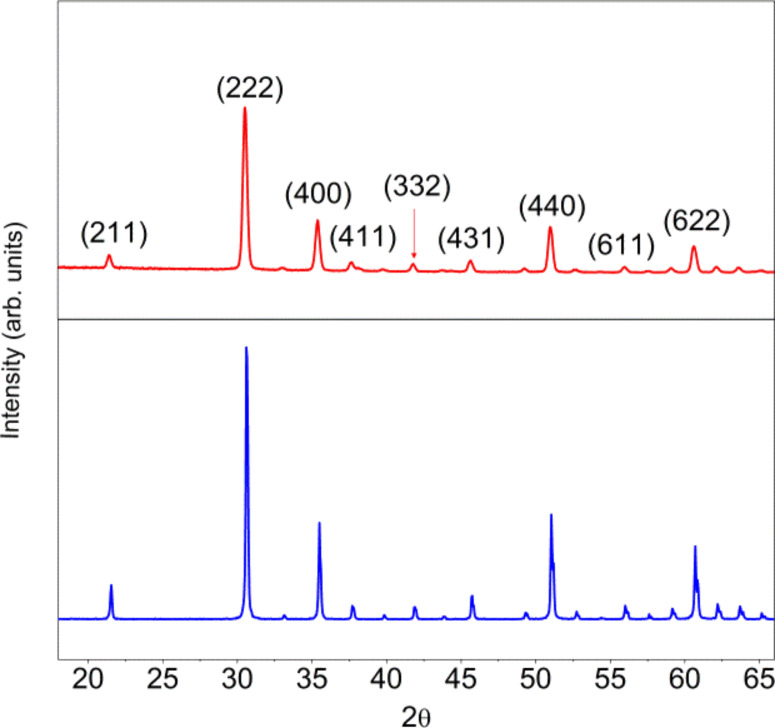
XRD patterns of the pure In_2_O_3_ octahedra (top) and a commercially available In_2_O_3_ powder (bottom). Adapted from [19].

The crystalline phase was investigated by means of X-ray diffraction (XRD). As shown in Figure 2, the as-grown samples show the typical features of cubic In_2_O_3_. The XRD pattern for a commercially available, cubic phase indium oxide has been added for comparison. No peaks belonging to other materials or impurities were found.

### Gas sensing analysis

#### Static sensor operation

**Figure 3:**
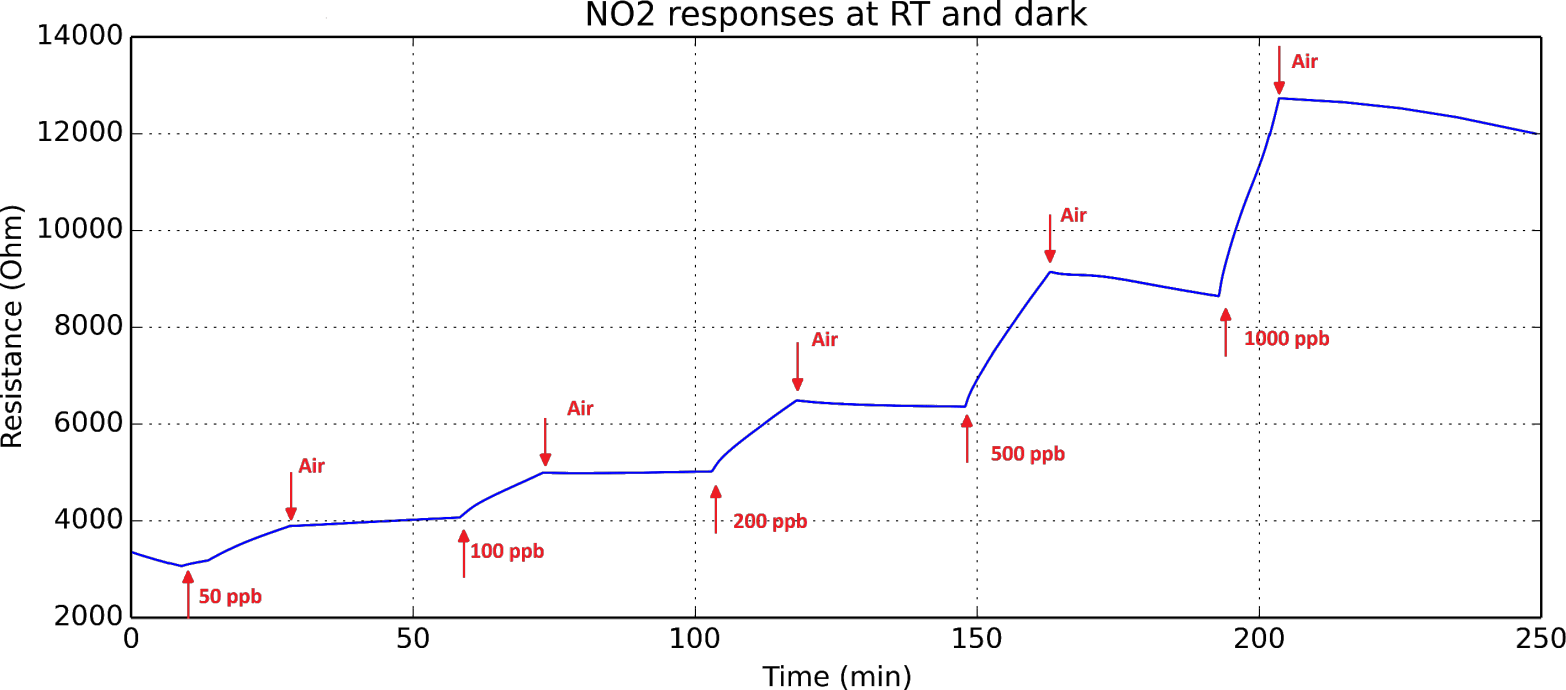
Successive response and recovery cycles during exposure to increasing concentrations of nitrogen dioxide of an indium oxide sensor operated at room temperature. No UV irradiation was applied during these tests. Reproduced from [19].

At first, the response of the nanomaterial to nitrogen dioxide was measured when the sensor was operated at room temperature (22 °C) and in the absence of UV light. Under these conditions, the sensor response was weak and not reversible. Figure 3 shows the evolution of the resistance of the sensor for response and recovery cycles to successively increasing concentrations of nitrogen dioxide that ranged between 50 ppb and 1 ppm. The duration of gas exposure and cleaning cycles was set to 15 and 30 min, respectively. The sensor resistance monotonically increases with the nitrogen dioxide concentration and when the sensor is flushed with pure dry air during the cleaning phases, its baseline resistance is never regained. Nitrogen dioxide is an oxidizing species. When a nitrogen dioxide molecule gets adsorbed on the indium oxide, it traps electronic charge from the conduction band of the nanomaterial (which is an *n*-type semiconductor), which results in an increase in the resistance of the sensor. This ionosorption process is a thermally activated process and room temperature is rather low, which explains the weak intensity of response to nitrogen dioxide under these conditions. Furthermore, at such a low operating temperature, most of the ionosorbed nitrogen dioxide molecules remain attached to the surface on indium oxide during the cleaning phases with dry air, which results in irreversible changes in sensor resistance.

**Figure 4:**
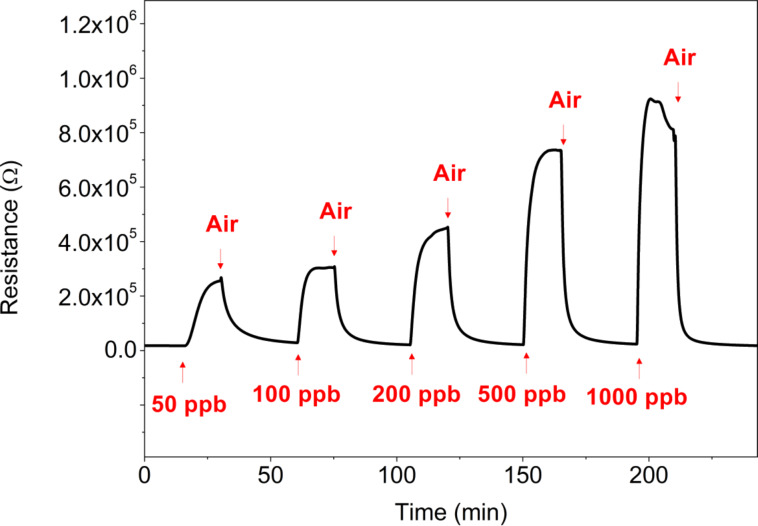
Successive response and recovery cycles during exposure to increasing concentrations of nitrogen dioxide of an indium oxide sensor operated at 130 °C. No UV irradiation was applied during these tests. Reproduced from [19].

In the second step, the sensor was operated at temperatures well above room temperature by driving a constant current through its resistive heating element. Figure 4 shows response and recovery cycles during exposure to increasing concentrations of nitrogen dioxide when operated at 130 °C and without UV irradiation. Once more, the durations of gas exposure and cleaning cycles were set to 15 and 30 min, respectively. Different operating temperatures between 100 and 250 °C were investigated and it was found that, when operated at 130 °C, the sensor showed the highest response to nitrogen dioxide. Furthermore, at this operating temperature, the sensing mechanism was fully reversible, since the sensor was able to regain its baseline resistance value during the cleaning phases. Sensor response, defined as the ratio between the sensor resistance in nitrogen dioxide and sensor resistance in dry air, ranged between 16.3 (50 ppb) and 60 (1 ppm). Response and recovery times, defined as the time needed to reach 90% of resistance change varied with nitrogen dioxide concentration between 4 and 8 min. Sensitivity to nitrogen dioxide, defined as the slope of the calibration curve in the range from 50 to 1000 ppb was 0.046 ± 0.004 ppb^−1^.

**Figure 5:**
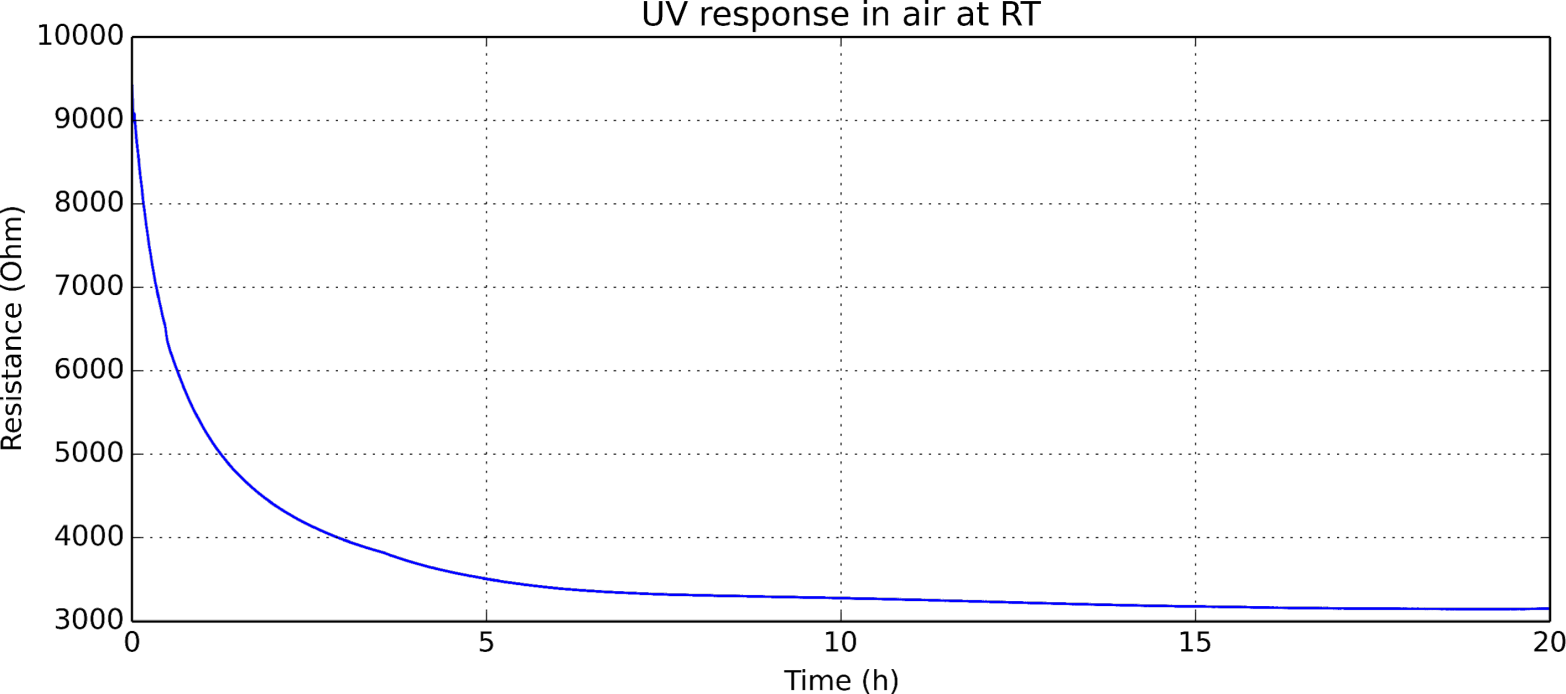
Resistance change of an indium oxide sensor suddenly exposed to UV light (the UV diode is switched on at time *t* = 0). The heating element of the sensor is not used in this test.

Prior to employ UV activation for the detection of nitrogen dioxide, the effect of a sudden irradiation of the indium oxide nanomaterial with UV light was investigated. For this, the evolution of sensor resistance was monitored when a UV diode was switched on at *t* = 0. No current was driven through the heating element of the sensor during this test. The evolution of sensor resistance is shown in Figure 5. Upon exposure to UV light, there is a significant drop in sensor resistance. This resistance change tails off over many hours indicating that the sudden exposure to UV light results in the triggering of changes in the nanomaterial that have slow dynamics. The decrease in sensor resistance can be explained by different mechanisms.

- Electrons in the valence band of indium oxide absorb energy from the UV light to jump to the conduction band. The bandgap of cubic indium oxide is about 3 eV and the 325 nm UV diode employed (3.8 eV) should allow these transitions to occur. Such transitions decrease the resistance of an n-type semiconductor film.
- UV light has been reported to act as a cleaner of the surface of metal oxides by promoting the desorption of adsorbed surface species. When an indium oxide sensor is in the presence of oxidizing species (e.g., oxygen, nitrogen dioxide), UV irradiation will result in the lowering of the amount of oxygen and/or nitrogen dioxide adsorbates. Since indium oxide is an *n*-type semiconductor, the desorption of oxidizing species from its surface translates into an increase in the number of free charge carriers and, thus, in a decrease in the DC resistance of the gas sensor [18–19].
- Furthermore, the partial removal of oxygen adsorbates may trigger the diffusion of bulk oxygen towards the surface, especially when the UV irradiated metal oxide sensor is operated at temperatures well above room temperature [20].

These mechanisms are reversible and when the UV light diode is switched off, the sensor eventually returns to its original baseline resistance value. However, the third mechanism has rather slow dynamics and may be responsible for the long time needed (few hours) for stabilizing the DC resistance of a sensor operated at constant temperature, after a sudden change in UV irradiation.

**Figure 6:**
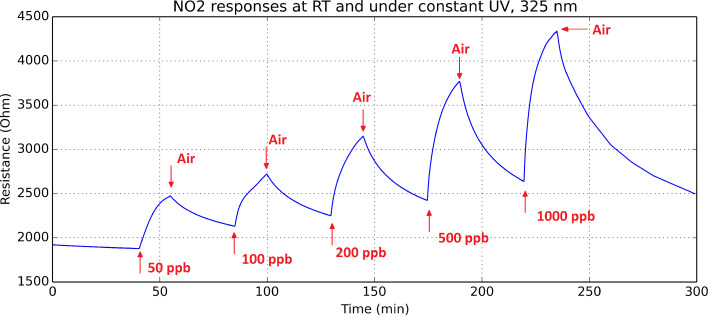
Response and recovery cycles of an indium oxide sensor exposed to different concentrations of nitrogen dioxide under constant UV light. The heating element of the sensor is not used in this test. The duration of exposure and recovery cycles was set to 15 and 30 min, respectively. Adapted from [19].

The UV diode was switched on and the sensor was left for 8 h under a flow of pure dry air. Then, the response and recovery of the sensor to different concentrations of nitrogen dioxide was studied under constant UV irradiation, without driving current through the sensor heating element. Figure 6 shows these response and recovery transients.

Unlike when the sensor is operated at room temperature without UV irradiation, under constant UV light, the sensor tends to regain its baseline resistance. However, response intensity to nitrogen dioxide is smaller and response and recovery dynamics are slower than when the sensor is operated at 130 °C without UV irradiation. In fact, the recovery phase in every response/recovery cycle shown in Figure 6, which is set to 30 min, is not long enough for the baseline to be fully regained. This behaviour can be explained as follows. UV light helps desorbing ionosorbed species from the surface of indium oxide [18]. Therefore, during the cleaning phases in which the sensor is flushed with dry air, UV light helps desorbing nitrogen dioxide adsorbates, reducing the indium oxide and decreasing the resistance of the sensor. During the response phases, there is a competition between the ionosorption of new nitrogen dioxide molecules from the ambient onto the surface of indium oxide and the desorption of nitrogen dioxide adsorbates caused by UV irradiation. The equilibrium concentration of nitrogen dioxide adsorbates depends on the ambient concentration of nitrogen dioxide, which results in increasing sensor resistance values for increasing concentrations of this pollutant gas. Table 1 summarises and compares the detection of nitrogen dioxide under these two different operation modes (i.e., constant temperature and constant UV irradiation). From the results in this table, it can be derived that operating the sensor at 130 °C is more favourable than operating it under constant UV irradiation for detecting nitrogen dioxide.

**Table 1:** Comparison of sensor response intensity, response and recovery times for an indium oxide sensor under two different operating conditions.

	response to NO_2_, 500 ppb (*R*_NO2_/*R*_air_)	response time (min)	recovery time (min)

sensor operated at 130 °C, UV diode off	47	4.5	5.3
sensor operated under constant UV light, heating element off	1.8	≈15	>30

#### Dynamic sensor operation

**Figure 7:**
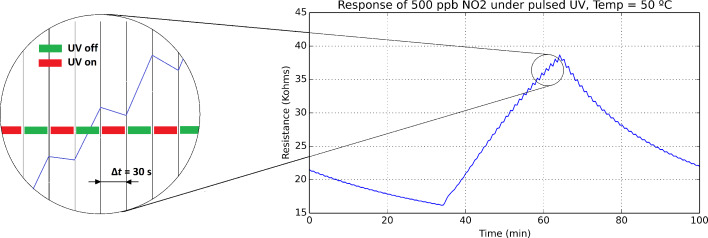
Successive recovery, response (nitrogen dioxide, 500 ppb) and recovery cycles of an indium oxide sensor under pulsed UV light. The heating element of the sensor is not used in this test. The inset shows an enlarged area of the evolution of sensor resistance in the presence of nitrogen dioxide. While the overall trend is for the resistance to increase due to the ionosorption of nitrogen dioxide molecules on the surface of indium oxide, locally, resistance decreases appear during the semi-period periods (30 s) in which the UV light is switched on.

The effect of UV irradiation on sensor response was further studied under a dynamic operation mode. In that case, during response and recovery cycles, the UV diode was periodically switched on and off by employing a square driving current signal with a period set to 1 min, while the sensor was operated at a given constant temperature (room temperature, 50 °C or 100 °C). Both in the presence of nitrogen dioxide (detection phase) or in pure dry air (cleaning phase), the sensor resistance increases while the UV diode is switched off and decreases while the UV diode is switched on, which results in the overall sensor response presenting a ripple. This ripple appears because UV light reduces indium oxide, which tends to re-oxidise when UV light is switched on. This can be seen in Figure 7, in which a sensor is operated at room temperature under pulsed UV light.

**Figure 8:**
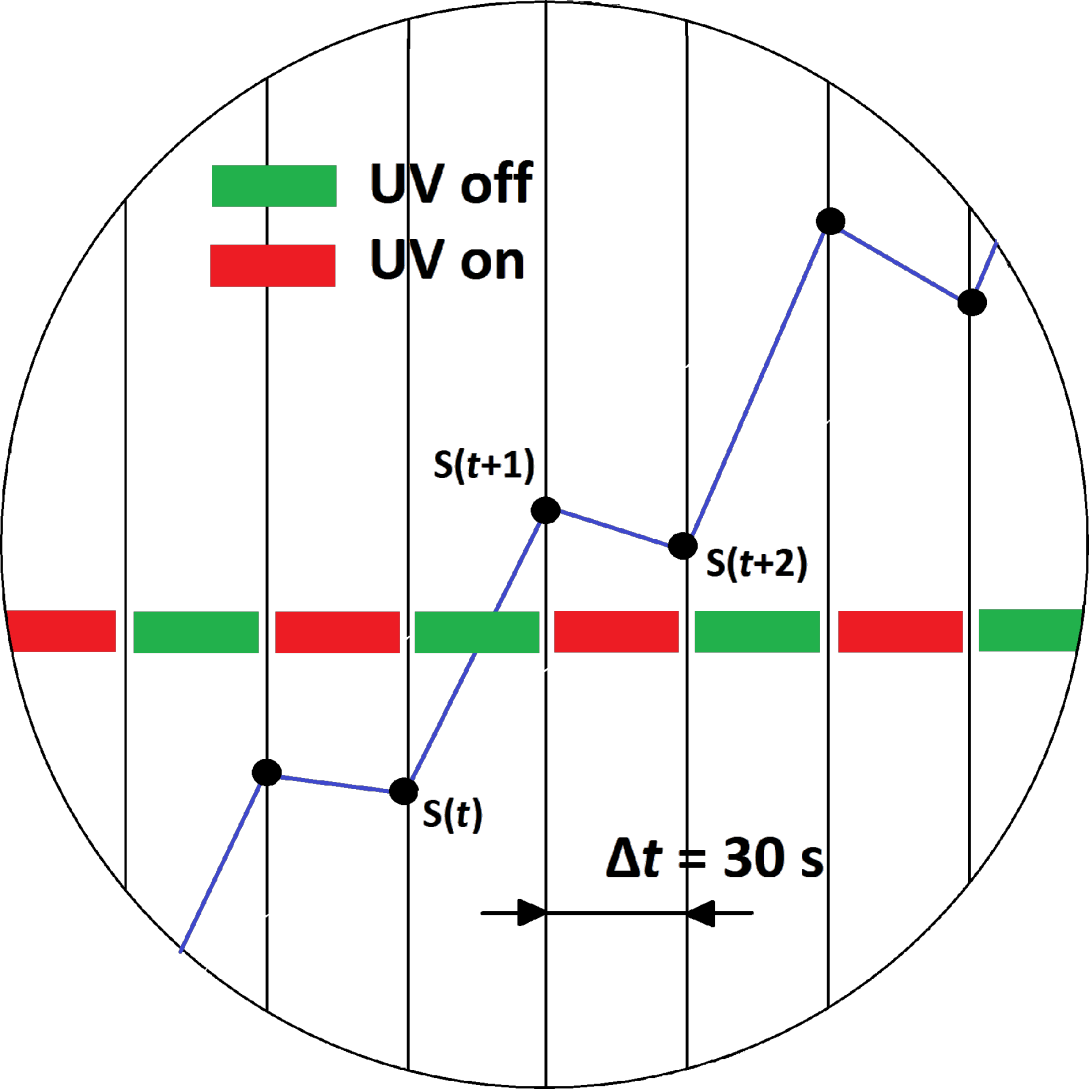
The instantaneous oxidation and reduction rates are defined as *R*_oxi_ = [*S*(*t* + 1) − *S*(*t*)]/Δ*t* and *R*_red_ = [*S*(*t* + 2) − *S*(*t* + 1)]/Δ*t*, respectively. Where *S* is the curve of the evolution of sensor resistance and Δ*t* is the semi-period in which the UV diode is kept switched on or off (Δ*t* = 30 s).

The dynamics of UV pulsed operation have been studied by computing the evolution of reduction and oxidation rates. The instantaneous reduction rate is computed as the local derivative of the resistance response curve during a semi-period in which the UV diode is switched on. Similarly, the instantaneous oxidation rate is the local derivative of the response curve during a semi-period in which the diode is switched off. Figure 8 illustrates how these two rates are computed.

**Figure 9:**
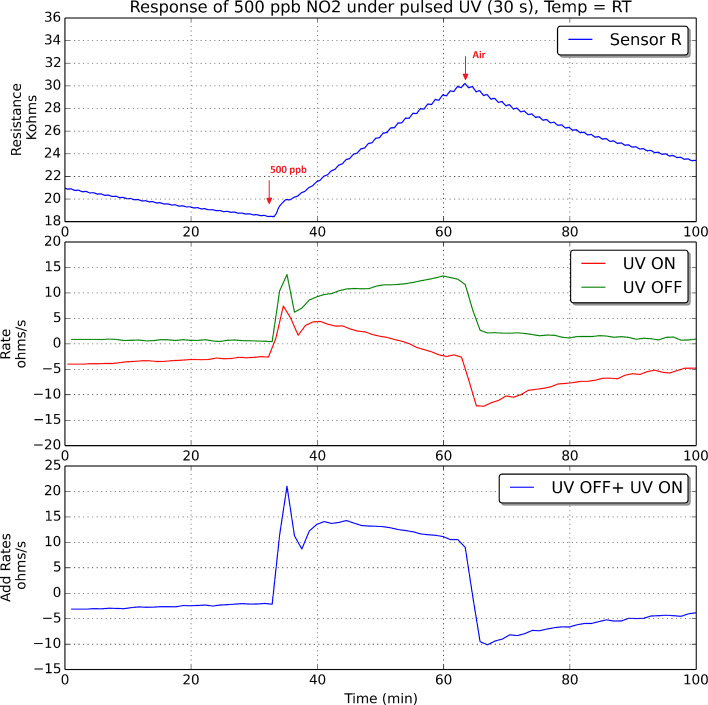
Indium oxide sensor response under pulsed UV irradiation. The sensor was operated at room temperature. Every panel shows the change in sensor resistance (top), the evolution of oxidation and reduction rates, which are plotted in green and red, respectively (middle), and the addition of these two rates (bottom) during the detection and recovery from an exposure to 500 ppb of nitrogen dioxide.

**Figure 10:**
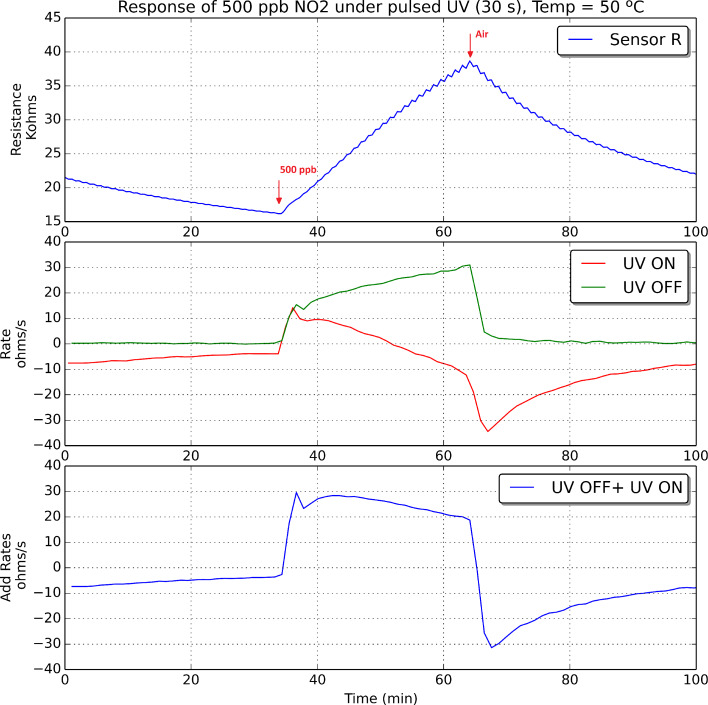
Indium oxide sensor response under pulsed UV irradiation. The sensor was operated at 50 °C. Every panel shows the change in sensor resistance (top), the evolution of oxidation and reduction rates, which are plotted in green and red, respectively (middle), and the addition of these two rates (bottom) during the detection and recovery from an exposure to 500 ppb of nitrogen dioxide.

**Figure 11:**
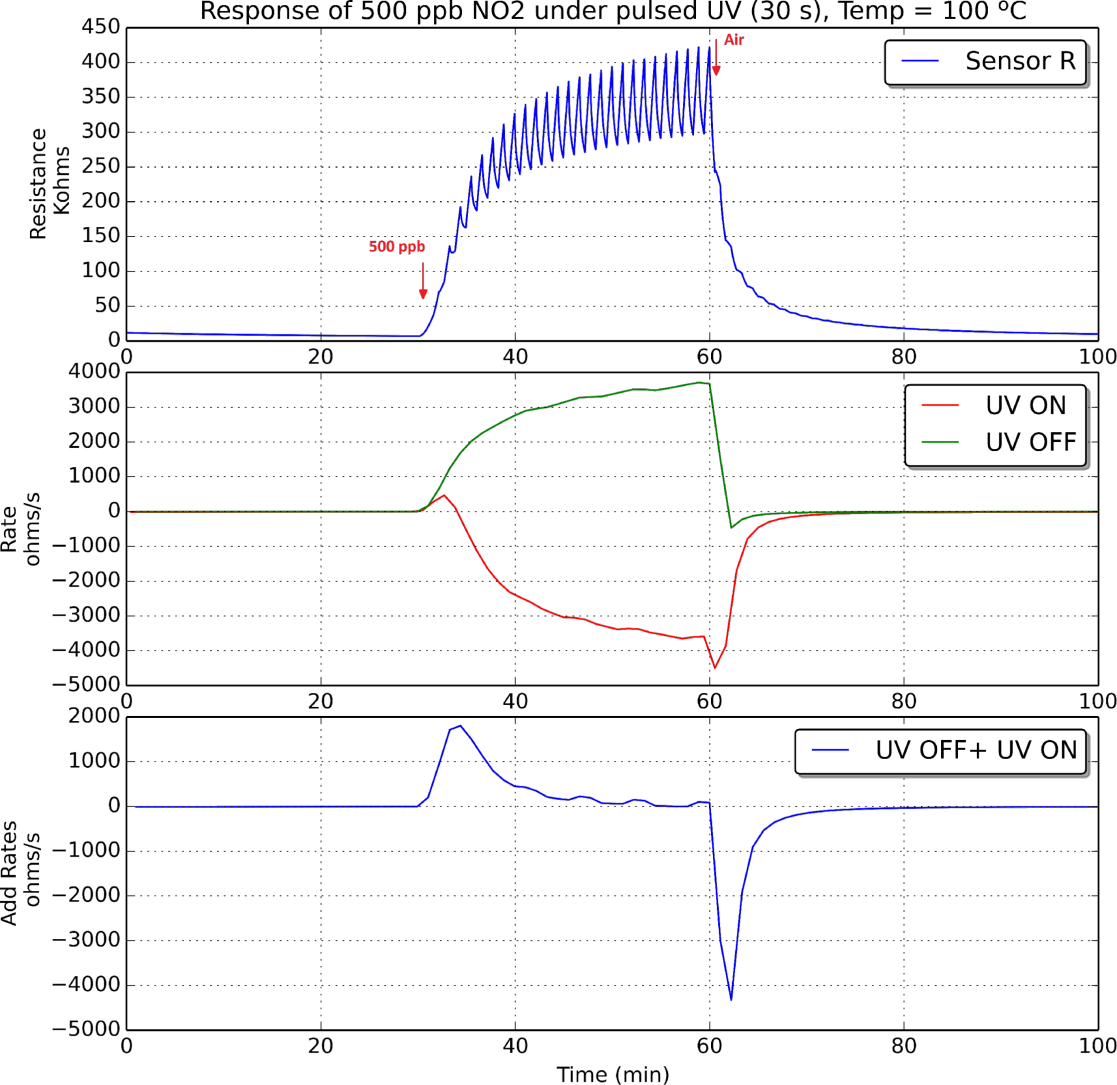
Indium oxide sensor response under pulsed UV irradiation. The sensor was operated at 100 °C. Every panel shows the change in sensor resistance (top), the evolution of oxidation and reduction rates, which are plotted in green and red, respectively (middle), and the addition of these two rates (bottom) during the detection and recovery from an exposure to 500 ppb of nitrogen dioxide.

The oxidation and reduction rates were computed for an indium oxide sensor operated at room temperature, 50 °C and 100 °C, both during nitrogen dioxide detection and recovery events. Figures 9–11 show these results when 500 ppb of nitrogen dioxide were measured.

According to what is shown in Figures 9–11, it can be derived that increasing the operating temperature of the sensor results in an increase in the rates of oxidation and reduction. If we consider now the rate of reduction (in red in Figures 9–11), its value should be negative if the surface of the sensor is reduced during the semi-period in which the UV diode is on. However, this is not the case during the initial period after a sudden change from pure air to nitrogen dioxide diluted in air. During a time period after the injection of nitrogen dioxide, there is a fast increase in the number of nitrogen dioxide molecules ionosorbed on the surface of indium oxide, which becomes more oxidised, and this in spite of the surface cleaning effect of UV irradiation. Soon after this initial action, the rate of adsorption of new nitrogen dioxide molecules decreases and the rate of reduction becomes negative. In other words, after the initial action, during the semi-period in which the UV diode is on, the amount of ionosorbed nitrogen dioxide molecules diminishes and sensor resistance decreases. The duration of this initial action decreases if the operating temperature of the sensor increases.

At the beginning of a cleaning phase in which nitrogen dioxide is suddenly removed from the ambient of the sensor, the rate of reduction peaks at higher negative values. This is because there is a combined effect of cleaning the surface of indium oxide with UV light and flowing the sensor with pure dry air, which results in a faster rate of desorption of nitrogen dioxide adsorbates. After this initial action, as the surface becomes cleaner, the rate of reduction tails off.

**Figure 12:**
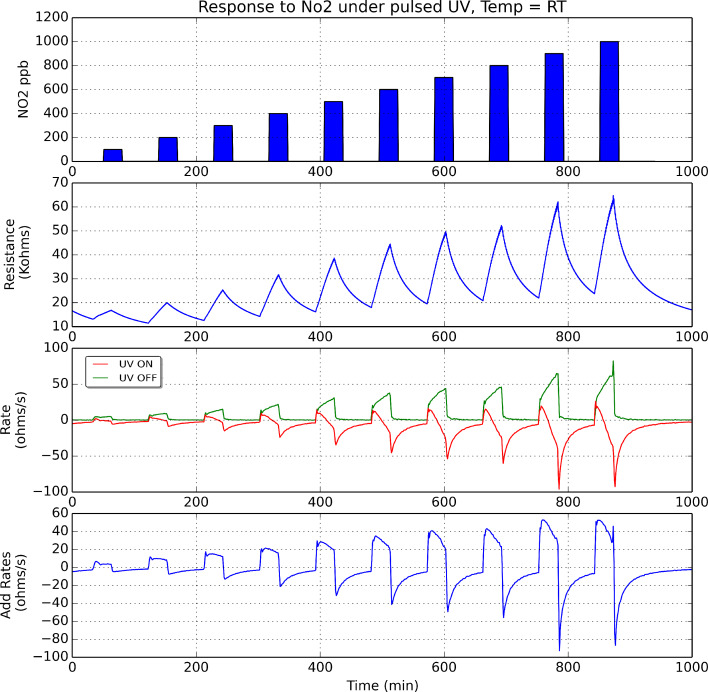
Analysis of the response of an indium oxide sensor operated at 50 °C under pulsing UV light. The upper sub-plot shows the pulses of increasing nitrogen dioxide concentrations (ranging from 100 to 1000 ppb) measured. The second sub-plot shows the dynamic response of the sensor (evolution of its resistance). The third sub-plot shows the evolution of the rates of reduction (in red) and oxidation (in green), which were computed as illustrated in Figure 8. The lower sub-plot shows the addition of the rates of reduction and oxidation.

If we consider now the rate of oxidation (in green in Figures 9–11), its value is positive and increases while nitrogen dioxide is present in the ambient of the sensor. When nitrogen dioxide is removed during a cleaning phase, the value of the oxidation rate decreases to zero. This is what it could be expected, because the rate of oxidation is computed when the UV diode is on. Finally, the curves appearing in the third sub-panel in Figures 9–11 show the addition of the rates of oxidation and reduction. It can be noted that these new curves are characterised by the presence of a maximum that appears at the initial stage of the transition between clean air and air in which nitrogen dioxide is diluted. Figure 12 shows the results of an experiment in which a sensor is exposed to successive response and recovery cycles to increasing concentrations of nitrogen dioxide while being operated at 50 °C and under pulsed UV light (pulse duration is 30 s). The addition of the rates of oxidation and reduction shows an initial maximum, the height of which is clearly related to the concentration of nitrogen dioxide. This peak appears after each transition from pure air to air containing nitrogen dioxide.

**Figure 13:**
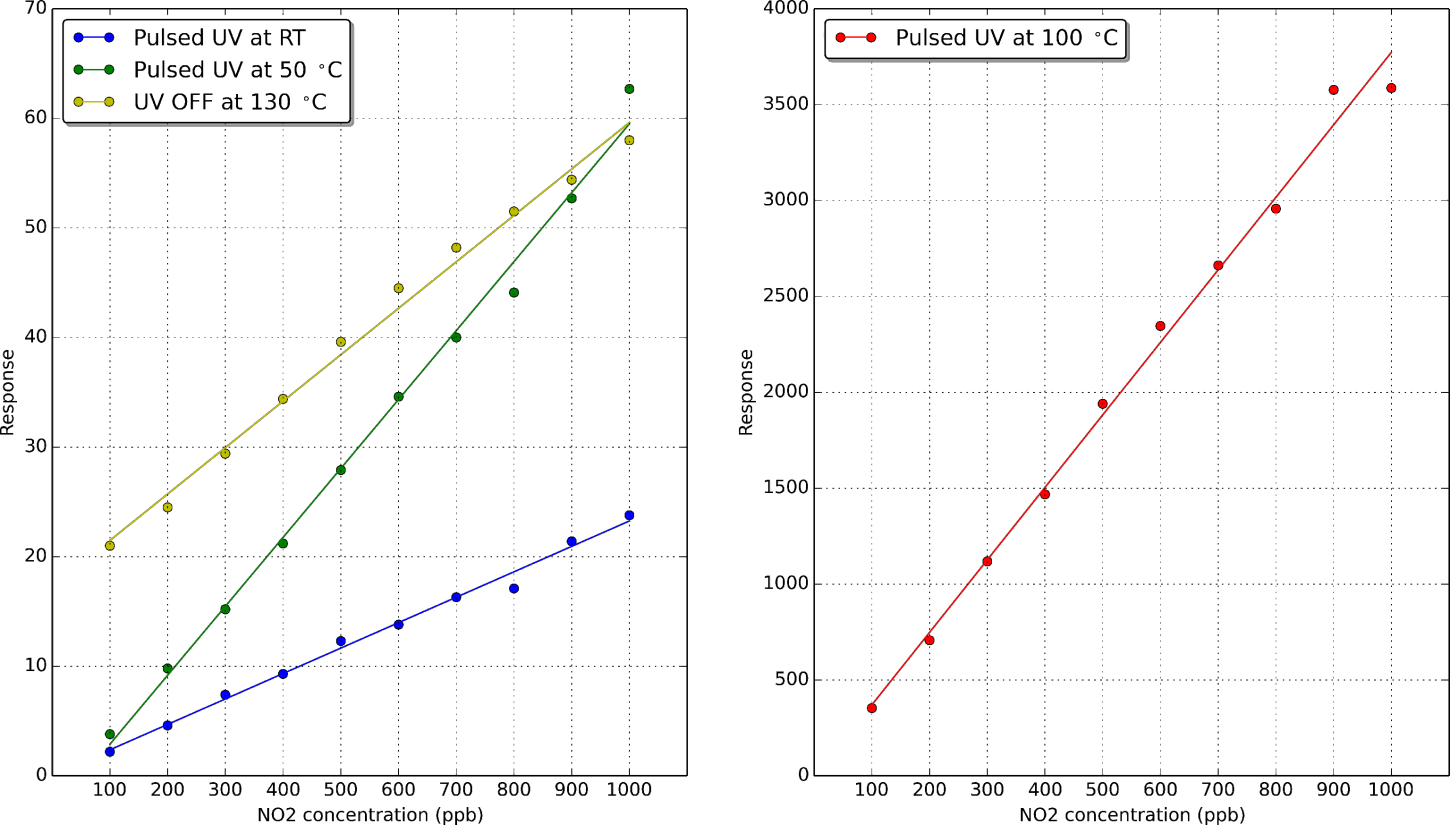
Calibration curves for the detection of nitrogen dioxide with an indium oxide sensor operated at three different temperatures under pulsed UV light. The calibration curve (light green) that appears in the left hand panel corresponds to the sensor operated at 130 °C and without UV activation. Response is either the ratio *R*_NO2_/*R*_air_ when sensors are operated at 130 °C without UV activation or, the sum of oxidation and reduction rates (in Ω/s) when sensors are UV activated.

Figure 13 shows the calibration curves obtained for the sensor response. The sensor response was measured as the intensity of the maximum of the addition curve (i.e., the sum of rates of reduction and oxidation) as a function of nitrogen dioxide concentration. These curves show that there is a quite linear behaviour of the response for the range of nitrogen dioxide concentrations measured and also that the sensitivity (i.e., slope of these calibration curves) increases with the operating temperature. In addition, to better assess the results obtained under pulsed UV light, the calibration curve of the sensor operated at 130 °C without UV activation is also shown in the left hand panel of Figure 13 (light green). For any of the values reported in Figure 13, the uncertainty (variance associated to the different sensors tested and replicated measurements performed) remains below 16%.

The sensitivity and response time to nitrogen dioxide of the indium oxide sensor under pulsed UV irradiation where computed. Sensitivity is generally defined as the change in response intensity for a given change in gas concentration. Given the fact that the calibration curves are linear, sensitivity is the slope of the calibration curves shown in Figure 13. Response time was defined as the time needed, after the injection of nitrogen dioxide, for the maximum to appear in the curve of the addition of reduction and oxidation rates (see Figure 12). These results are summarised in Table 2. In this table, the values for sensitivity and response time when the sensor is operated at 130 °C and without UV irradiation have also been included for better assessing the results obtained under pulsed UV light.

**Table 2:** Comparison of response time, sensitivity to nitrogen dioxide and power consumption for an indium oxide sensor under different operating conditions. Power consumption includes the power supplied to the heating element and/or the power supplied to the UV diode where relevant.

temperature	UV	response time (min)	sensitivity	power consumption

RT	pulsed	9.4	0.023^a^	41 mW
50 °C	pulsed	7.1	0.063^a^	151 mW
100 °C	pulsed	4.3	3.78^a^	560 mW
130 °C	off	4.5	0.046^b^	855 mW

^a^In Ω/s·ppb^−1^; ^b^in ppb^−1^.

From the values reported in Table 2, it can be derived that the combined heating and pulsed UV activation of the indium oxide sensor is the best approach for enhancing the detection of nitrogen dioxide. When the sensor is operated at 50 °C under pulsed UV light, there is nearly a 40% increase in sensitivity to nitrogen dioxide and an over 80% reduction in power consumption (in comparison to the standard constant temperature operation of the sensor without UV activation). This is reached at the cost of increasing response time. However, if the sensor is operated at 100 °C under pulsed UV light, there is an 80-fold increase in sensitivity and there is still a 35% saving in power consumption. In this case, response time is similar to the one observed when the sensor is operated at a constant temperature of 130 °C without UV irradiation. The figures reported about power savings need to be considered with care, since our ceramic transducer has not been optimised for power consumption. However, most of commercially available metal oxide gas sensors still use ceramic substrates and their power consumption is in the range of few hundred milliwatts. Therefore, the UV pulsed operation would still help reducing overall power consumption.

## Conclusion

The effects of heating and UV irradiation on the response toward nitrogen dioxide of screen-printed sensors employing vapour-phase transport synthesized indium oxide octahedra as active material have been studied. It was found that constant UV irradiation and no heating was unsuitable for reversibly detecting nitrogen dioxide with enough sensitivity. On the other hand, by combining mild heating (100 °C) with pulsed UV light irradiation of the sensor surface, resulted in a dramatic enhancement in sensitivity (up to an 80-fold increase) combined to the possibility of making significant savings in power consumption (at least 35% reduction) in comparison to the standard heated operation. This has been achieved by exploiting the dynamics of sensor response under pulsed UV light (i.e., the rates of oxidation and reduction of the indium oxide), which convey important information for the quantitative analysis of nitrogen dioxide. In the near future, further studies are envisaged to fully optimise the combined heating and UV pulsing operating mode of metal oxide gas sensors and their integration in semi-passive, flexible polymeric RFID tags. The integration of gas sensors with UV LEDs would increase overall cost. However, this cost could be a fraction of those incurred when producing a standard MOX sensor, provided that UV-activated sensors were produced in big numbers. This could be the case if such sensors were to integrate widespread personal or indoor air monitors.

## Experimental

The indium oxide nano-octahedra were grown onto silicon substrates via a vapour-phase transport method using a horizontal chemical vapour deposition (CVD) furnace. Substrates were cleaned before their insertion in the CVD reactor by three consecutive, five minutes long steps of sonication in acetone, ethanol and deionized water, respectively. Finally cleaned substrates were dried using a flow of pure dry air. In a typical synthesis, 0.3 g of high purity In metal powder (99.99% pure) purchased from Sigma Aldrich, was placed on an alumina boat. The distance between the alumina boat and the silicon substrate was 1 cm and these were placed at the centre of the furnace. Temperature was raised to 900 °C at a rate of 15 °C/min, and kept constant for 120 min. The indium oxide nano-octahedra were synthesized in a dynamic Ar atmosphere (300 mL/min) via a vapour–solid mechanism, since there was not need to pre-treat the silicon substrate by depositing catalysts. Indium vapours were generated from the In powder, which reacted with residual oxygen present in the furnace, thus forming oxidized clusters. When the temperature further increases, the oxidized In clusters act as nucleation centres for the formation of Indium oxide crystals. It has been reported that at growth temperatures between 800 and 1000 °C pyramids and octahedra can be obtained [18]. The facets exposed correspond to the most energetically stable atomic planes in the lattice. The furnace was left to cool down to room temperature and a light green film was found to coat the silicon substrate. The crystalline phase and morphological structural features were studied by means of X-ray diffraction (XRD, Bruker-AXS D8-Discover diffractometer with parallel incident beam) and field emission scanning electron microscope (FESEM, Jeol 7600F).

For SEM analysis, samples were coated with a 3 nm thick carbon layer to avoid charging effects.

Sensors were produced by employing a screen-printing technique. The as-synthesized nano-octahedra were mechanically removed from the silicon substrate employing a razor blade and mixed in a solution of 1,2-propanediol to form a printable ink. This ink was screen-printed on top of commercially available (Ceram Tech GmbH, Germany), alumina transducer elements, which comprised a pair of Pt interdigitated electrodes (front side) and a Pt heating resistor (back side). Each electrode comprises 8 arms (300 µm in width) with an electrode gap of 300 µm. The electrodes cover an area of 2.5 × 2.5 mm^2^. The heating element on the backside of the substrate had a resistance of 8 Ω at room temperature. Once printed, sensors were left for an hour to level and then were dried at 150 °C. Finally they were fired at 300 °C for 12 h. The active films were 6 µm thick, quite porous and with a surface roughness of about 2 µm [21]. The largest dimension of indium oxide octahedra is about 500 nm. Therefore, the UV light can reach the surface of a significant amount of the nanomaterial. In total three sensors were produced and tested.

**Figure 14:**
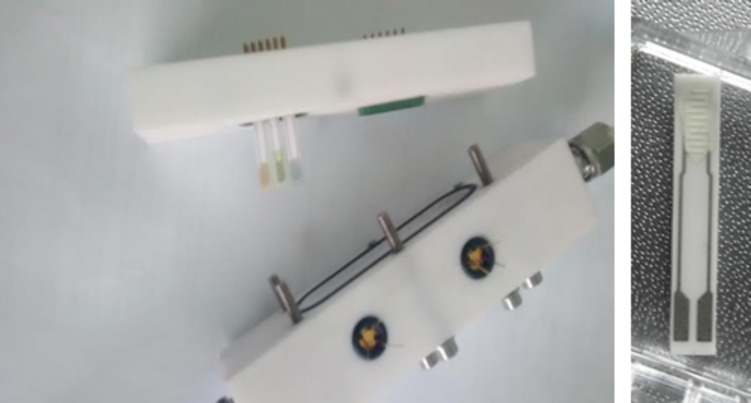
Sensor chamber used during the experiments, which can house up to six sensors (left). The chamber includes sockets for the UV diodes. Picture of an indium oxide gas sensor (right).

A sensor test chamber was designed and constructed in Teflon. Its inner volume was 24 cm^3^. The chamber contains sockets to which up to six sensors can be plugged in to be tested. The cover lid houses two UV LEDs so sensors can be operated in ‘temperature mode’ when a constant current is driven through their heating element while the UV diodes are off; in ‘UV activation mode’ when heaters are not used and the UV LEDs are on; and in ‘mixed mode’ when both heating elements and UV LEDs are used simultaneously. The LED to sensing film distance was set to 12 mm, which considering the radiation aperture of the LEDs used (120°), ensured achieving a homogeneous UV irradiation of the sensors. The UV LEDs employed were manufactured by SETI, Sensor Electronic Technology Inc., Columbia, SC, USA [22] (model UVTOP320TO39FW) and their maximal emitting optical power was 400 µW at 325 nm. The specifications of the UV LED indicate that to avoid saturation, the drive current should be kept below 30 mA at an ambient temperature under 55 °C. The drive current was pulsed (diode was on 50% of the time only) and limited to 15 mA. Furthermore, the LED was placed 2 cm away from the heated area of the sensor and kept inserted in a thermally insulating housing. Therefore, no saturation effect affected our experimental set-up. A picture of the sensor chamber and of a gas sensor is shown in Figure 14.

Gas mixing and delivery to the test chamber was performed by using a set of computer controlled mass-flow meters. A calibrated gas cylinder of nitrogen dioxide diluted in synthetic dry air and a zero-grade synthetic air gas cylinder were employed to deliver different, reproducible concentrations of nitrogen dioxide at constant flow of 100 sccm.
